# Pulmonary Alveolar Proteinosis with Respiratory Failure-Anaesthetic Management of Whole Lung Lavage

**Published:** 2009-06

**Authors:** Sunita Nandkumar, Madhavi Desai, Manju Butani, Z Udwadia

**Affiliations:** 1 Senior consultant, P.D. Hinduja National hospital & Medical research centre, Veer savarkar marg, Mahim, MUMBAI-400016, INDIA; 2 Associate Consultant, P.D. Hinduja National hospital & Medical research centre, Veer savarkar marg, Mahim, MUMBAI-400016, INDIA; 3 Senior consultant & HOD, P.D. Hinduja National hospital & Medical research centre, Veer savarkar marg, Mahim, MUMBAI-400016, INDIA; 4 Consultant, Chest physician, P.D. Hinduja National hospital & Medical research centre, Veer savarkar marg, Mahim, MUMBAI-400016, INDIA

**Keywords:** Pulmonary alveolar proteinosis, Whole lung lavage, Broncho-pulmonary lavage, Anaesthesia

## Abstract

**Summary:**

Pulmonary alveolar proteinosis (PAP) is a rare disorder characterized by accumulation of amorphous acellular phospholipid material in the lungs. Whole lung lavage is the standard therapy which gives dramatic clinical improvement and offers a long term survival to these patients.

A 43-year-old man suffering from PAP presented to casualty with NYHA grade IV dyspnoea with oxygen saturation (SaO_2_) on pulseoximetry 67% on room air and 78% with O_2_ 6 L/min. He underwent whole lung lavage under general anaesthesia using one lung ventilation with 37 F left end bronchial double lumen tube. The lung lavage was initially performed for the left lung and for the right lung 4 days later. The patient was discharged home with oxygen saturation of 96 % on room air.

## Introduction

Pulmonary alveolar proteinosis (PAP), first described in 1958 by Rosen et al^1^, has proved to be a rare but interesting disorder resulting in accumulation of amorphous lipoproteinaceous acellular material in lungs following impaired clearance of surfactant by macrophages. Transgenic murine models have clearly established that hematopoietic growth factor (granulocyte-macrophage colony stimulating factor [GM-CSF]) is critical for local regulation of surfactant homeostasis. Additional human studies have linked the presence of a circulating, neutralizing anti–GM-CSF antibody in adults with idiopathic PAP. Therefore, in the current paradigm, there are three major clinical subtypes of PAP: the most common adult idiopathic variety, which is pre-sumably autoimmune (with a circulating anti–GM-CSF antibody); a neonatal variety that is likely due to a defect in surfactant proteins B or C or the common ß-chain of the GM-CSF receptor; and secondary PAP associated with occupational exposures or immunologic disorders. Preliminary reports from several groups indicate that a subset of adult patients with idiopathic PAP respond favorably to GM-CSF therapy.^1^ Epidemiological data is scarce but one estimate suggests an annual incidence of the order of 2 to 5 per million. The long term outlook for the patients with PAP has greatly improved with use of whole lung lavage.

We report a case of PAP in a patient who was diagnosed 10 years ago. He had undergone whole lung lavage and then was asymptomatic for 10 years. He again presented in acute respiratory failure despite GM-CSF therapy and hence was subjected to whole lung lavage (WLL) which resulted in dramatic clinical and radiological improvement.

## Case report

A 43-year-old farmer, who was a known case of PAP presented to casualty with NYHA grade IV dyspnoea with respiratory rate 28/min, pulse 88/min, BP 100/68 mmHg, oxygen saturation (SaO_2_) on pulseoximetry 67% on room air and 78% with O_2_ 6 L/min. The trachea was intubated in casualty, ventilated with FiO_2_1, PEEP 5 cm H_2_O and a respiratory rate of 20 which resulted in peak airway pressure of 40cm H_2_O and SaO_2_ of 83% in intensive care unit (ICU). Radiography of chest revealed diffuse bilateral asymmetrical infiltrates which had worsened as compared to his previous X-rays. CT scan of the thorax showed bilateral patchy alveolar filling shadows with air bronchogram. The trachea was extubated after 48 hours of ventilation and the patient was put on FiO_2_ 0.6 under venturi mask with SaO_2_ of 88-89%. He was scheduled for whole lung lavage.

Patient's blood investigations showed a Hb 18.7 g%, haematocrit 53.3%, WBC count 75,000/cmm neutrophils 97%. 2D-Echocardigram showed left ventricular ejection fraction (LVEF) 60%, pulmonary artery pressure by jet 20-25 mmHg. The arterial blood gas(ABG) analysis was as follows: PH =7.467, PaO_2_ 52.1 with FiO_2_ 0.6, PaCO_2_ = 32.2, HCO_3_ =23. His blood pressure was 94/56mmHg and heart rate 50/min.

## Management

The whole lung lavage was carried out in the ICU in two sessions. The right internal jugular vein and right radial artery were cannulated under local anaesthesia prior to procedure. The CVP was 10-12 mmHg. After preoxygenataion for 5 minutes the patient was induced with intravenous midazolam 0.05mg Kg^−1^ + fentanyl 6mcgKg^−1^, 5 % sevoflurane via inhalation and iv succinylcholine 2 mgKg^−1^. The trachea was intubated with Broncho-cath 37 F (Mallinckrodt) left endobronchial double lumen tube (DLT) and ventilated with FiO_2_ 1 using Aestiva 5(Datex–Ohmeda) ventilator of the anaesthesia machine. Position of the DLT was confirmed with auscultation and using 2.8 mm (outer diameter) fiber-optic bronchoscope. Anaesthesia was maintained with midazolam 0.01 mgKg^−1^ hr^−1^ + fentanyl 1mcg.kg^−1^ hr^−1^, + atracurium 0.4 mg.kg^−1^. hr^−1^ infusion and isoflurane 0.2-1.5%. Dopamine 5mcg.kg^−1^ min^−1^ was started along with the induction in view of his pre-procedure haemodynamic status.

ABG analysis was done immediately after induction while both lungs were ventilated with FiO_2_ 1 when PaO_2_ was 65.1mmHg. Since we decided to lavage the left lung first, it was clamped and degassed, while the right lung was ventilated with FiO_2_ 1 + PEEP 5 cm H_2_O for next 20 minutes. At the end of 20minutes ABG was repeated which showed a PaO_2_ of 52.5 mmHg (Table-1). Since patient tolerated the 20 minutes test of one lung ventilation haemodynamically and maintained PaO_2_ fairly, Broncho-pulmonary lavage(BPL) on left lung was performed.

**Table 1:** ABG analysis during left lung lavage

	PH	PaCO2	PaO2	HCO3	SaO2	K	Na	FiO2
Pre-procedure	7.41	33.1	59	25	90	3.4	133	0.6
Rt lung 20 min OLV	7..33	56	52.5	28.5	84	3.4	134	1+PEEP5
End of procedure	7..21	60	50.1	24	78	4.2	134	1+PEEP5
Both lungs ventilation	7..24	52	110.5	21.9	97	4.2	134	1+PEEP5
16 hrs post ventilation	7..39	32	85	19	96	3.4	134	0.8
Post extubation	7.44	32.8	62	22	92	3.4	136	0..5

**Fig 1:**
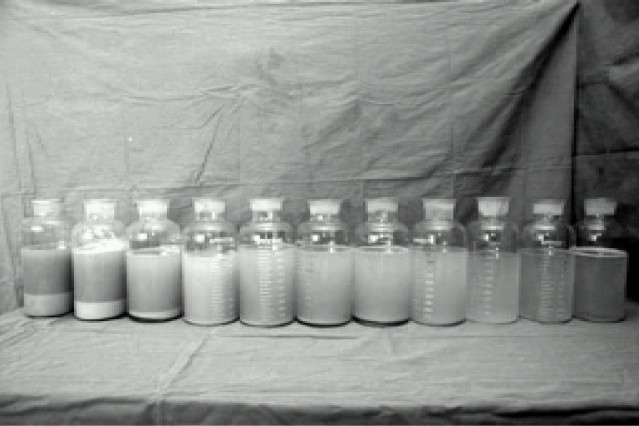
Effluent lavage fluid

Lavage was performed using 1000 ml – 1200 ml aliquots of normal saline warmed to body temperature; that was rapidly instilled into the left lung. Chest percussions were performed for several minutes while the fluid was indwelling to help loosen the proteinacious material. The saline was then withdrawn from the degassed lung. The saline was infused and drained by gravity using wide bore tubing with a Y – shape connection. Lavages were continued until the effluent saline became clear and nonviscous (Fig-1). This required 10 cycles of lavage with 10.5 L saline. Throughout the procedure invasive blood pressure, CVP, urine output, SaO2, capnography, rectal temperature, repeated ABG and serum electrolytes were monitored meticulously. Lowest SaO_2_ in the lavage was 80%, maximum PaCO_2_ 60 mm Hg and blood pressure was maintained 95/60- 120/70 mm Hg with dopamine of 5mcg.kg^−1^ min^−1^. After re-expansion of lavaged lung with 100% O_2_ the DLT was replaced with standard 8.5 Portex cuffed single lumen endotracheal tube at the end of the procedure. The procedure took 4&½ hours with total affluent saline of 10.5 L, effluent fluid 9.0 and urine output 750 ml. Patient remained intubated and ventilated for an additional 16 hours to permit clearing of secretions and the trachea got extubated the next morning. After extubation the ABG showed PaO_2_ 61, SaO_2_ 92% with FiO_2_ 0.5 (Table-1), he was maintaining BP 100/60 mmHg off dopamine, hence was shifted to ward from ICU.

This patient then underwent right lung lavage after 4 days with 17 L of normal saline with same anaesthetic management using Broncho-cath 37 F (Mallinckrodt) left endobronchial DLT. The procedure was conducted after performing a 20 minutes test of OLV of left lung. This time SaO_2_ could be maintained at 90% and above throughout the procedure. (Table-2) There were less haemodynamic, temperature and metabolic fluctuations during this lavage. The procedure took 6&½ hours with total affluent saline of 17 L in 15 cycles, effluent fluid 15.5L and urine output 850 ml. Like the previous lavage, DLT was replaced with Portex cuffed single lumen endotracheal tube at the end of the procedure and the trachea was extubated the next morning after a good chest toilet and overnight ventilation.

**Table 2:** ABG analysis during right lung lavage

	PH	PaCO2	PaO2	HCO3	SaO2	K	Na	FiO2
Pre-procedure	7.47	31.8	87	23.3	95	3.6	136	0.5
Lt lung 20 min OLV	7..34	52	65	28.5	91	3.3	134	1+PEEP5
End of procedure	7..32	48.4	69	24.3	92	3.1	137	1+PEEP5
Post extubation	7.44	32.8	62	22	92	3.4	136	0..5
24 hr Post extubation	7.44	33.0	89	24	96	3.4	136	0..35

## Discussion

Bronchopulmonary lavage, introduced by Ramirez in late 1960s is still the gold standard therapy for PAP^1^ and is usually indicated in PAP only for patients with significant symptoms and hypoxemia. With the patient under general anaesthesia, the lungs are usually lavaged one at a time with 3 to 5 days between lavages. The technique has improved over years enhancing effective removal of accumulated material in lungs. It has been recently shown that recombinant Granulocyte-Macrophage colony stimulating factor (GM-CSF) 5μg kg^−1^ day^−1^ for 6-12 weeks appears to benefit a subset of adult patients with PAP and may represent alternative to whole lung lavage in treating this disease.^1^,^2^ Few cases are reported to be benefited by plasmapheresis.^1^,^3^ Our patient had received GM-CSF therapy before reaching our institute but had failed to respond clinically. His WBC count was 75,000/mm^3^ with neutrophils 97% following GM-CSF therapy.

It is advised to lavage the more affected lung first for allowing better lung to provide gas exchange. Left lung is lavaged first in equal involvement of both lungs, leaving the larger right lung to support gas exchange during OLV^4^. We conducted BPL on left lung first as both the lungs of this patient were equally affected.

Preoxygenation for 5 minutes before induction is extremely important, not merely as a part of routine protocol; but because failure of denitrogenation of the lung leaves nitrogen bubbles in alveoli and limits the effectiveness of lavage.

Traditionally the lavage is conducted in lateral decubitus to lavage dependent lung and to ventilate the nondependent lung to minimize the risk of spillage of saline to other lung. But this is associated with higher ventilation perfusion mismatch in a already severely hypoxic patient Therefore, unlike the previous reports Beccaria et al^5^ ventilated dependent lung and lavaged the nondependent one to give better ventilation perfusion ratio. Andrew Perez and colleagues have performed last part of the lavage in prone position specifically to clear the posterior segment of lungs. In their study of six patients, right lung lavage of one patient had to be terminated because of displacement of DLT in prone.^6^ We performed the procedure in supine position with lateral tilt using a wedge to elevate the ventilated lung as it was conducted in the cumbersome ICU cubicle and not on the usual operating room table, and patient being somewhat haemodynamically unstable requiring inotropes.

Before starting lavage a 20 minutes test of one lung ventilation was performed with high inspiratory oxygen fraction up to 1 and low positive end- expiratory pressure of 5 cm H_2_O while the other lung was actively deflated and degassed as much as possible in order to reproduce the worst possible condition that could occur during lavage. In this way safety of the procedure was tested before proceeding to lavage. Recently a less complicated procedure, bronchofiberscopic lobar lavage under local anaesthesia has been proposed for carefully selected patients. Cheng and colleagues underline the fact that this procedure is safe and does not require anaesthetic support, however it is recommended only in milder disease or conversely in particularly severe cases in which the physiological derangement of whole lung lavage especially during drainage phase would not be tolerated by patient which is revealed after 20 minutes test of one lung ventilation^4^,^7^. In critically ill patients extra-corporal membrane oxygenation can be used to support gas exchange during lavage.^4^,^8^

Infusion of large volumes of saline in lungs is associated with mediastinal shift, increased intrathoracic pressure, CVP, Pulmonary capillary wedge pressure, arterial oxygen tension and hypotension. Despite the increase in CVP and PCWP it has been postulated that ventricular filling is not increased, and may be actually compromised following impaired venous return to the thorax of unknown etiology.^9^ Arterial oxygen tension increases during filling because blood flow to nonventilated lung is decreased by the lavage fluid infusion pressure. Hypotension, rise in CVP and rise in SaO_2_ were classically noticed in our patient.

Associated special problems during the procedure are displacement of the DLT and a leak through the DLT resulting in spillage of lavage fluid into the ventilated lung. That is why it is mandatory to confirm the placement of the DLT by fiberoptic broncho-scope. The procedure has to be terminated immediately and the DLT can be replaced with single lumen ETT in case of sudden displacement. Continuous monitoring to detect the loss of lung isolation was done as follows:

- The appearance of bubbles in the lavage fluid draining from the lavaged side.
- Increased resistance to ventilation and appearance of rales and rhonchi on the ventilated side.
- A fall in arterial oxygen saturation and rise in end tidal carbon dioxide due to spillage leading to inadequate ventilation.

During the procedure, all the measures should be taken to avoid hypothermia since the procedure takes several hours to complete and with use of lavage fluid of ambient temperature. Temperature of the patient was maintained using lavage fluid warmed to body temperature, warming blanket and i.v. fluid warmer.

Though post lavage radiographs show partial clearing of PAP by 6 weeks compared with baseline pre-wash radiograph, pulmonary mechanics and gas exchange improve rapidly after the lavage.^10^ SaO_2_ of our patient improved from 92% with 0.5 FiO_2_ to 96% on room air in four days after the lavage and was discharged home.

Our case report illustrates that the rare disorder of Pulmonary Alveolar Protienosis can be successfully managed with technique of Whole Lung Lavage with a good teamwork between anaesthesiologist, chest physician, respitatory therapiest and intensivist.
